# Sonographic twinkling artifact for diagnosis of acute ureteral calculus

**DOI:** 10.1007/s00345-019-02773-z

**Published:** 2019-04-24

**Authors:** Ningning Liu, Yue Zhang, Kun Shan, Rui Yang, Xuening Zhang

**Affiliations:** 1 Department of Urolithiasis Treatment Center, Second Hospital of Tianjin Medical University, Tianjin, 300211 China; 2 Department of Nuclear Medicine, Second Hospital of Tianjin Medical University, Tianjin, 300211 China; 3 Department of Radiology, Second Hospital of Tianjin Medical University, Hexi Pingjiang Road No. 23, Tianjin, 300211 China

**Keywords:** Sonographic twinkling artifact, B-ultrasound, CT, Ureteral calculus, Renal colic

## Abstract

**Objective:**

We compared the performance of color Doppler twinkling artifacts with B-ultrasound and computed tomography (CT) for diagnosis of ureteral calculus in patients with acute renal colic.

**Methods:**

The location and size of ureteral stones in 2268 patients with acute renal colic were determined using the two ultrasound methods and CT. All cases were followed up for 2–8 weeks.

**Results:**

Color Doppler twinkling artifacts had a sensitivity of 96.98%, specificity of 90.39%, positive predictive value (PPV) of 99.77%, and negative predictive value (NPV) of 41.23%. B-Ultrasound had a sensitivity of 96.39%, specificity of 80.77%, PPV of 99.53%, and NPV of 34.43%. CT had a sensitivity of 99.59%, specificity of 94.23%, PPV of 99.86%, and NPV of 84.48%. The area under the receiver operating characteristic curve was 0.925 for color Doppler twinkling artifacts, 0.863 for B-ultrasound, and 0.963 for CT.

**Conclusion:**

For the diagnosis of ureteral calculus, the sonographic twinkling artifact had a similar performance as CT. We suggest use of the sonographic twinkling artifact instead of CT for patients with acute renal colic to reduce the examination time and exposure to radiation, and to provide earlier access to treatment.

## Introduction

Urolithiasis is one of the most common diseases of the urinary system. Although the prevalence and incidence of urolithiasis vary throughout the world, urolithiasis has become increasingly more common over the past few decades. For example, during the past 30 years the prevalence in North America increased from 7 to 13%, the prevalence in Europe increased from 5 to 9%, and the prevalence in Asia increased from 1 to 5% [1]. During the past 10 years, the incidence of urolithiasis in China increased from 4 to 6.4%, and the prevalence in men is currently twice that of women [2–4]. Recurrence of urolithiasis is also common. For example, the recurrence rate after 1 year is 6–17%, the recurrence rate after 3–5 years is 21–53%, and the lifetime recurrence rate is 60–80% [5, 6].

**Fig. 1:**
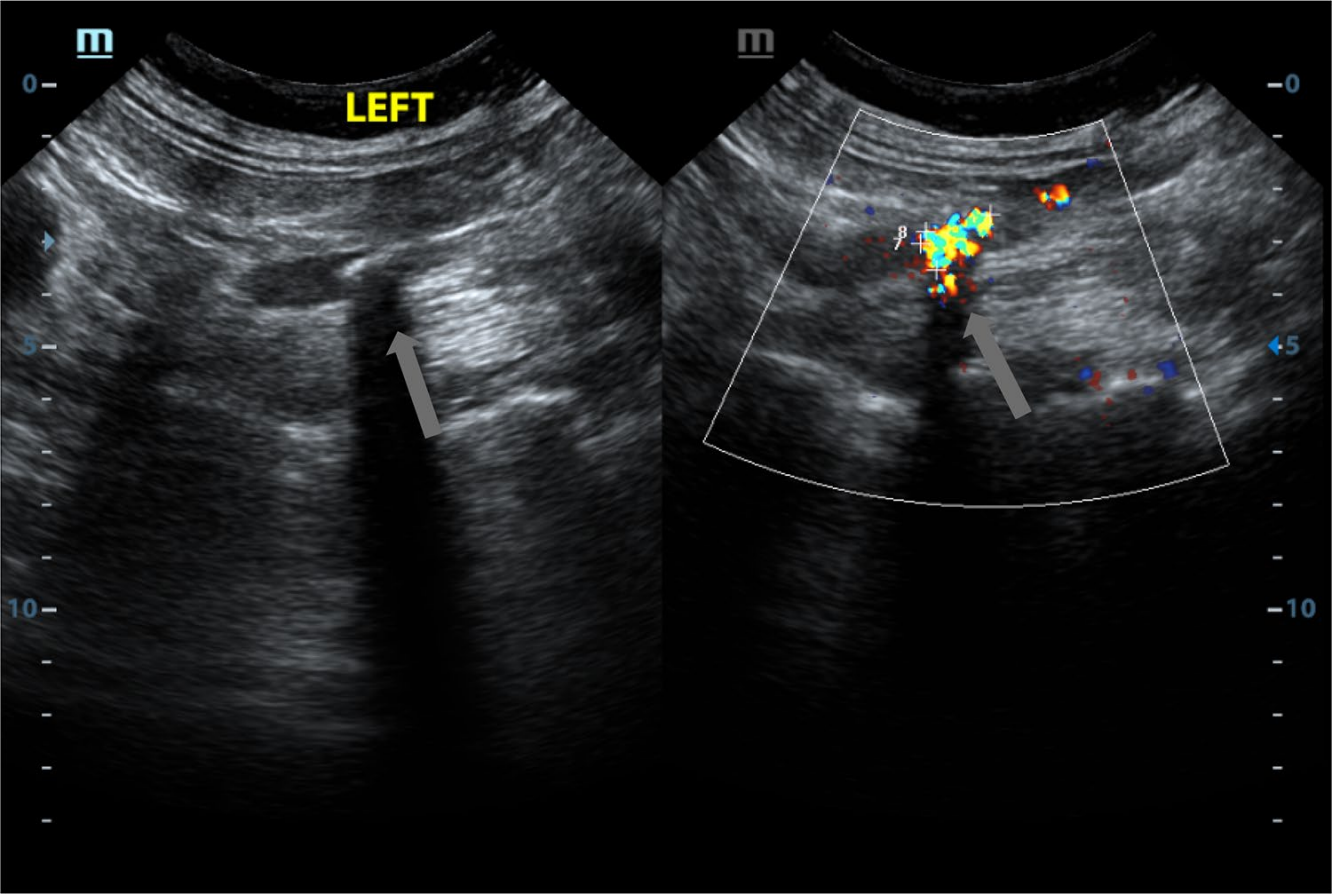
Representative upper ureteral calculus visualized using B-ultrasound (left) and color Doppler twinkling artifacts (right). The B-ultrasound indicated an uneven echo of the stone, and an unclear boundary. The color Doppler twinkling artifacts covered the stone, and the boundaries were clearer

Because of the high morbidity and recurrence rates of urolithiasis, it is important to use economic, safe, fast, and accurate methods for examination and diagnosis. Computed tomography (CT) has high sensitivity and specificity and is widely regarded as the best imaging method for assessing acute renal colic. However, patients and doctors are increasingly concerned about the risks associated with radiation exposure when CT is used for the repeated evaluation of acute renal colic and for the draining of stones. Therefore, it is likely that ultrasound without ionizing radiation will replace CT in the diagnosis of acute renal colic. The sensitivity and specificity of ultrasound in the diagnosis of urinary calculus are lower than those of CT, and the diagnosis also depends on the clinician’s interpretation. However, the “twinkling artifact” of color Doppler ultrasound may increase the performance of ultrasound in the diagnosis of urolithiasis. This twinkling artifact in color Doppler flow imaging is characterized by rapidly changing red and blue color signals behind a strong reflective material [7–9] (Fig. 1).

Although the cause of twinkling artifact is unclear, studies have increasingly used sonographic twinkling artifacts to improve the accuracy of detecting urinary calculus [10, 11]. In our experience, for a patient with severe pain, flatulence, and urinary tract irritation, the sonographic twinkling artifact may be able to quickly confirm a diagnosis of urolithiasis, allowing rapid symptomatic treatment and resolution of pain.

This study aimed to improve the accuracy of diagnosing patients experiencing acute renal colic pain due to ureteral calculi and provide a reliable diagnostic basis for further treatment by comparing the use of the color Doppler sonographic twinkling artifact with B-ultrasound for the diagnosis of acute ureteral calculus.

## Materials and methods

### Study population

This was a prospective study of 2268 adult patients (≥ 18 years-old) with renal colic who were admitted to the Urology Department of Tianjin Medical University Second Hospital from October 2017 to July 2018. This study was approved by the Ethics Committee of Tianjin Medical University, and each participating patient agreed to provide a medical history and participate in the urological examination. Immediately after a patient reported pain relief, CT and ultrasound examinations of the urinary system were performed. All patients diagnosed with ureterolithiasis using CT were included. Exclusion criteria were: recent history of urological disease (tumor, urinary tract infection, and other diseases that may affect the diagnosis of ureteral calculus or confuse the symptoms of ureteral calculus), bilateral lumbar and abdominal pain, pregnancy, and suspected urinary tract infection (≥ 10 white blood cells per high-power field).

### Methods

Two doctors, each with more than 5 years of experience in ultrasonography of the kidney, ureter, and bladder, performed color Doppler ultrasound and B-ultrasound on each patient using the IU-22 (Philips, convex array probe, 3.5 MHz) and the DC-8S (Mindry, convex array probe, 3.5 MHz). For these examinations, the patient was in a supine position with both hands on the chest, and the abdomen was completely exposed. First, the presence of hydronephrosis and perirenal effusion was determined on the affected side. Then, the operator scanned down the ureteropelvic junction to the junction of the ureter and the bladder to observe the expansion, extent, and shape of the ureter and confirm the diagnosis. Regardless of bladder filling, the sonographic twinkling artifact and gray-scale ultrasound were used to determine the presence of stones in the ureteral lumen. During the scanning process, the focus was placed slightly deeper than the stone, and the gain setting was controlled. The diagnostic criterion for ultrasound is a strong echo in the ureteral lumen of the affected side, with or without sound shadow and hydronephrosis on the affected side.

After the ultrasound examination, the urinary system was scanned using the GE Light Speed Pro 64-row helical CT. The scanning layer thickness and interval were each 5 mm, and scanning ranged from the bilateral upper pole to the pubic symphysis.

All images were stored in a computer, and examined by two radiologists (each with more than 10 years of experience) who were blinded to the final diagnosis and the statistical analysis. All patients with ureteral calculi based on ultrasound were followed up for 2–8 weeks. The gold standard for the diagnosis of ureteral calculus is ureteroscopic calculus removal or discharge during urination.

### Statistical analysis

Data were analyzed using MedCalc 18.2 (MedCalc, Ostend, Belgium) and SPSS 20.0 (SPSS, Chicago Illinois, USA). Each quantitative indicator is presented as mean ± SD and a paired *t* test was used to compare different groups. The diagnostic performance of each method was assessed by measuring its sensitivity, specificity, positive predictive value (PPV), negative predictive value (NPV), and area under the receiver operating characteristic curve (ROC). A *P* value below 0.05 was considered significant.

## Results

### General characteristics of patients

We examined the records of 2268 patients with emergency renal colic, 2216 of whom had diagnoses of urinary calculus based on ureteroscopic calculus removal or stone discharge (Table 1). The other 52 patients had appendicitis (*n* = 4), a gynecological disease (*n* = 16 cases), or a urinary tract infection (*n* = 32).

**Table 1:** Characteristics of enrolled patients (*n* = 2268)

Variable	*n* (%) or mean ± SD
Male	1789 (78.9%)
Female	479 (21.1%)
Age (years)	48.2 ± 13.7
BMI (kg/m^2^)	27.6 ± 7.5
Ureteral stone position	
Bilateral	3 (0.1%)
Right side	1019 (46.0%)
Left side	1194 (53.9%)
Upper section	793 (35.8%)
Middle section	569 (25.7%)
Lower section	854 (38.5%)
Ipsilateral hydronephrosis	
None	264 (11.9%)
Mild	1919 (86.6%)
Moderate	33 (1.5%)
Stone size (cm) from twinkling artifacts	0.9 ± 0.3
Stone size (cm) from B-ultrasound	0.9 ± 0.3
Stone size (cm) from CT	1.0 ± 0.2
Bladder volume (mL)	154.4 ± 43.4

Among patients with acute renal colic, 1789 cases (78.9%) were male and 479 (21.1%) were female, the mean age was 48.2 ± 13.7 years, and the mean BMI was 27.6 ± 7.5 kg/m^2^. Three patients had bilateral ureteral stones, 1019 had stones only on the right side, and 1194 cases had stones only on the left side. There were 1952 patients with hydronephrosis and 264 patients without hydronephrosis. Among the hydronephrosis patients, 1919 had mild hydrops and 33 had moderate hydronephrosis.

### Diagnostic performance of the three methods

A total of 2149 cases (94.8%) were positive for ureteral calculus based on the sonographic twinkling artifact, 2136 cases (94.2%) were positive based on B-ultrasound, and 2207 cases (97.3%) were positive based on CT. The sensitivity of these methods ranged from 96.39% (twinkling artifact) to 99.59% (CT), the specificity ranged from 80.77% (B-ultrasound) to 94.23% (CT), the PPV ranged from 99.53% (twinkling artifact) to 99.86% (CT), and the NPV ranged from 34.42% (B-ultrasound) to 84.48% (CT) (Table 2).

**Table 2:** Diagnosis of ureteral calculus by color Doppler twinkling artifacts, B-ultrasound, and CT

Method	Sensitivity (%)	Specificity (%)	PPV (%)	NPV (%)
Twinkling artifacts	96.98	90.39	99.77	41.23
B-ultrasound	96.39	80.77	99.53	34.43
CT	99.59	94.23	99.86	84.48

*PPV* positive predictive value, *NPV* negative predictive value

**Fig. 2:**
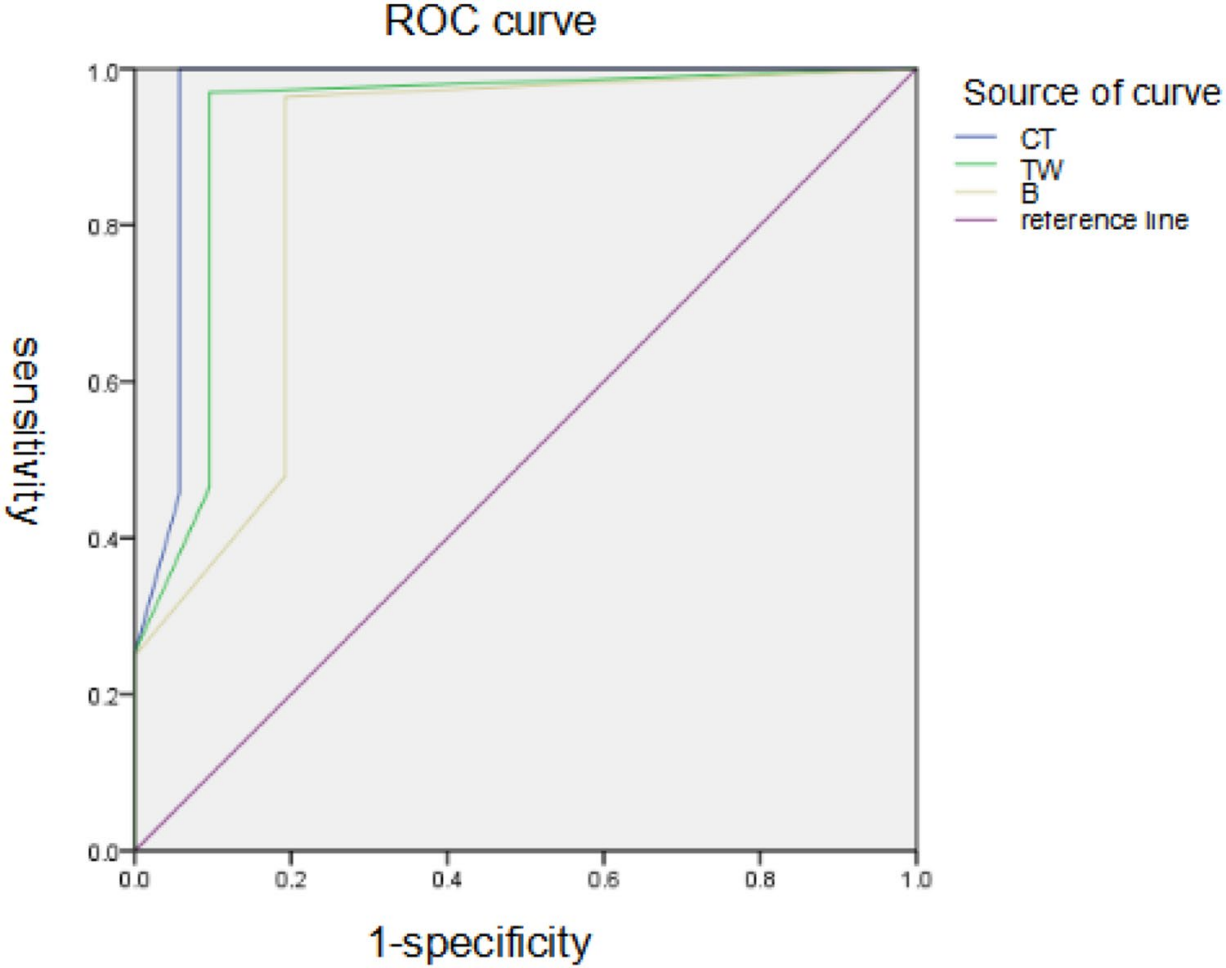
Receiver operating characteristic (ROC) curves for the diagnosis of ureteral calculus by color Doppler twinkling artifacts (TW), B-ultrasound (B), and CT. Twinkling artifacts, AUC = 0.925 (95% CI 0.913–0.935); B-ultrasound, AUC = 0.863 (95% CI 0.848–0.877); CT, AUC = 0.963 (95% CI 0.954–0.970)

Thus, the area under the curve (AUC) was 0.925 ± 0.026 for the twinkling artifact, 0.863 ± 0.034 for B-ultrasound, and 0.963 ± 0.021 for CT. Pairwise comparisons using a *t* test indicated these AUC values were significantly different (*P* < 0.05 for all comparisons) (Fig. 2, Table 3).

**Table 3:** Comparison of AUC determined by color Doppler twinkling artifacts, B-mode ultrasound, and plain CT

	AUC difference	SEM	95% CI	*P*
B-TW	0.062	0.026	0.011–0.112	**0.016**
B-CT	0.100	0.030	0.041–0.158	**0.001**
TW-CT	0.038	0.038	0.005–0.071	**0.025**

*SEM* standard error of the mean, *CI* confidence interval, *AUC* area under the cure

### Accuracy of measuring calculus size of the three methods

CT is currently the gold standard for diagnosis of urinary calculus and determination of stone size. Therefore, we compared stone size determined by different methods among the 2136 patients with true positive CT results. We calculated the difference in size of the ureteral calculus measured by the twinkling artifact and B-ultrasound as the absolute difference from the size determined by CT, and expressed this as ΔTW and ΔB, respectively. A paired *t* test indicated that the twinkling artifact provided a significantly better estimate of stone size than B-ultrasound (ΔTW = 0.001 ± 0.015 cm, ΔB = 0.0088 ± 0.115 cm; *t* = − 2.579, *P* = 0.010) (Table 4).

**Table 4:** Difference of ureteral calculus size determined by color Doppler twinkling artifacts and B-mode ultrasound relative to CT

	Mean	SD	SEM	95% CI	*T*	*P*
ΔTW − ΔB	− 0.006	0.115	0.002	− 0.002 to − 0.011	− 2.579	**0.010**

*SD* standard deviation, *SEM* standard error of the mean, *CI* confidence interval, *ΔTW* difference in size determined by scattering artifacts and CT, *ΔB* difference in size determined by B-ultrasound and CT

### Relationship of patient characteristics and ureteral calculus characteristics

**Fig. 3:**
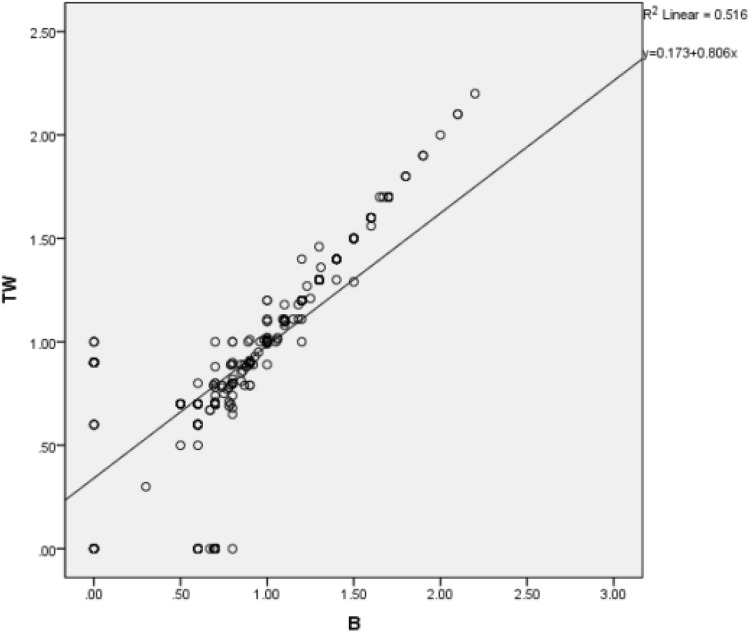
Correlation of stone size (cm) determined by color Doppler twinkling artifacts (TW) and B-ultrasound (*n* = 2136, *r* = 0.718, *P* < 0.001)

We also used multivariate linear regression to analyze the relationship between stone size and patient characteristics. In particular, we used sex, age, BMI, stone position, extent of hydronephrosis on the affected side, and extent of bladder filling as independent variables and stone size from the twinkling artifact and B-ultrasound as the dependent variables. The results indicate no significant relationship between stone size and any of the examined patient characteristics (*P* > 0.05 for all comparisons). However, there was a significant positive correlation (*r* = 0.718, *P* < 0.001) between stone size measured by the twinkling artifact and B-ultrasound (Fig. 3).

## Discussion

CT is considered the gold standard for diagnosis of ureteral stones. Accordingly, we found that CT had a sensitivity greater than 99% and a specificity greater than 94%. However, because the prevalence and recurrence of renal calculus have increased over time, many patients are subjected to repeated CT scanning for the detection of small stones, and experience a risk of radiation-related adverse effects [12–14]. Thus, as an economic and safe diagnostic tool, ultrasound plays an increasingly important role in the diagnosis of acute renal colic [15]. Recent studies reported that for the diagnosis of urolithiasis, ultrasound has a sensitivity of 90–93% and a specificity of 95–100% [8]. For example, a recent study of the diagnosis of ureteral calculus by ultrasound in 100 patients with suspected renal colic reported a sensitivity of 90%, a specificity of 100%, a PPV of 100%, and a NPV of 67% [8]. Our results indicated that B-ultrasound had a sensitivity of 96.39%, a specificity of 80.77%, a PPV of 99.53%, and an NPV of 34.43%.

Previous research indicated that use of color Doppler twinkling artifacts increased the sensitivity of gray-scale ultrasound from 45 to 99% [16]. Similarly, Mitterberger et al. compared the sensitivity of gray-scale ultrasound with color Doppler ultrasound in the diagnosis of 77 urinary calculi in 44 patients, and found that the gray-scale ultrasound had a sensitivity of 66%, but color Doppler twinkling artifacts had a sensitivity of 97% [10]. Our results indicate that use of color Doppler twinkling artifacts for diagnosis of ureteral calculus had a sensitivity of 96.98% and a specificity of 90.39%. Our sensitivity and specificity values were slightly lower than those of the previous studies, possibly because more of our patients had calculi in the middle and lower sections of the kidney. Patients with acute ureteral calculi in the middle and lower regions have greater difficulty in filling the bladder due to severe pain, and often have symptoms of urinary tract irritation. Intestinal flatulence and other symptoms could also make diagnosis more difficult in these patients.

Thus, the use of color Doppler twinkling artifacts is very effective for detection of renal calculus. A major advantage of this method is that the position of the stones can be determined in advance, thus shortening the time from diagnosis to treatment [7, 17, 18]. Stones that remain undetected by twinkling artifacts may have a rough surface or a unique chemical composition [19, 20].

Chelfouh et al. performed an in vitro color-flow sonography of 47 calculi and found that the sensitivity and specificity of calculus identification without twinkling artifacts were 60% and 83%, respectively [19]. Further analysis of calculi without twinkling artifacts showed that they were composed of calcium oxalate monohydrate [19]. However, these studies were performed in vitro, so in vivo confirmation is necessary because the distance between tissues reached by sound waves may cause attenuation and affect the generation of twinkling artifacts [6]. This previous study also found that twinkling artifacts were not associated with renal pelvis and ureter expansion, similar to the findings of Lee et al. [21]. Taken together, these results suggest that use of sonographic twinkling artifacts can provide an accurate diagnosis of ureteral calculi, and is a suitable alternative to posterior acoustic diagnosis of ureteral stones.

In daily clinical practice, B-ultrasound is not effective in identification of stones in emergency cases or when a mass has an echo suggestive of a stone but an unclear boundary. Use of the sonographic twinkling artifact can help determine the location, size, and boundary of a stone. Moreover, our ROC results indicate that the accuracy of the sonographic twinkling artifact is not inferior to that of CT, although color ultrasound is safer and more convenient than CT. Analysis of stone size also indicated that the results from sonographic twinkling were closer to those from CT than B-ultrasound. Therefore, the combined use of the sonographic twinkling artifact with B-ultrasound can improve the accuracy of ureteral calculus diagnosis, reduce the examination time, and facilitate early treatment. Although the sensitivity and specificity of the sonographic twinkling artifact and B-ultrasound are similar to those of CT, they had lower NPVs than CT. Thus CT has the advantage of diagnosing ureteral calculus as a gold standard.

## Conclusion

The sonographic twinkling artifact has a high diagnostic value in the detection of acute ureteral calculi, is better in the detection of ureteral stones than B-mode ultrasound, and is not inferior to CT for experienced radiologists who have more than 5 years of experience using this technique. We therefore suggest the increased use of sonographic twinkling artifacts for the early diagnosis of acute renal colic.

## Abbreviations

PPV: Positive predictive value
CT: Computed tomography
AUC: Area under the curve
NPV: Negative predictive value
ROC: Receiver operating characteristic curve
TW: Twinkling artifacts
